# Pregnancy and cardiovascular disease in developing countries of South Asia—A narrative review

**DOI:** 10.1038/s44294-025-00101-y

**Published:** 2025-10-07

**Authors:** Farhala Baloch, Alveena Zafar, Momina Faisal, Anoosha Marium, Lumaan Sheikh, Haleema Yasmin, Pamela Susan Douglas, Zainab Samad

**Affiliations:** 1 Section of Cardiology, Department of Medicine, Aga Khan University, Karachi, Pakistan; 2 Medical College, Aga Khan University, Karachi, Pakistan; 3 Department of Medicine, Aga Khan University, Karachi, Pakistan; 4 Department of Obstetrics and Gynecology, Aga Khan University, Karachi, Pakistan; 5 Department of Obstetrics and Gynecology, Jinnah Postgraduate Medical Center, Karachi, Pakistan; 6 Department of Cardiology, Duke University, Durham, USA

**Keywords:** Diseases, Health care

## Abstract

According to the World Health Organization, low-middle-income countries report higher maternal mortality rates in South Asia compared to higher-income countries. Pregnancy with cardiovascular diseases is a prominent contributor to these preventable deaths. Reasons behind poor maternal outcomes are multifactorial, including unstable healthcare systems, knowledge gaps, and sociocultural factors. This review discusses the concept of iceberg model, a tool for health system analysis to identify potential factors behind poor maternal outcomes.

## Introduction

**Fig. 1:**
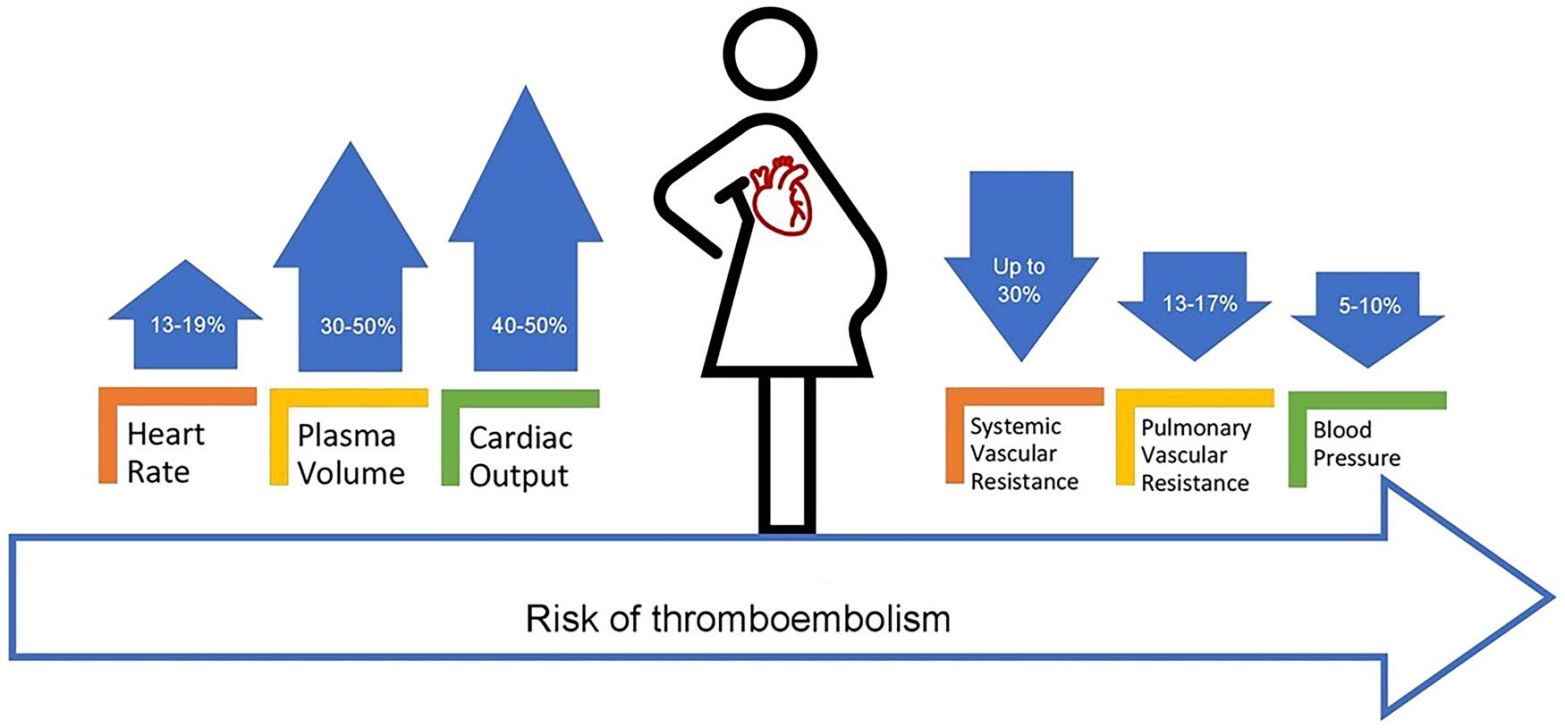
Cardiovascular changes in pregnancy. A diagrammatic illustration of physiological adaptations in the maternal heart during pregnancy.

The maternal cardiovascular system undergoes several physiological changes during pregnancy starting from the first trimester and continuing till delivery and post-partum period and sometimes beyond. These changes in the form of essential physiological adaptations are necessary to support both the mother and the fetus. Both functional and structural changes occur in the maternal cardiovascular system. These changes include an increase in plasma volume, cardiac output (CO) up to 40–50% above baseline, and an increase in heart rate, along with a decrease in systemic and pulmonary vascular resistance and blood pressure. In addition, due to its hypercoagulable nature, pregnancy poses a greater risk of thromboembolism to the mother. A healthy heart can tolerate these changes without compromising the mother or fetus’s health. However, in the presence of preexisting cardiac conditions, the mother and fetus are at greater risk of complication and poor outcome due to sub-optimal adaptation and the detrimental effect of physiological adaptation on the existing cardiac condition^1^ (Fig. 1).

The coexistence of pregnancy and cardiovascular diseases (CVD) poses pregnant women and healthcare providers with a unique challenge both in the developing and developed world. CVD complicates approximately 1–4% of pregnancies in developed countries^2^, and the adverse maternal outcomes rate is four times higher (up to 16%) in women with previously diagnosed cardiac conditions^2^. More than 50% of maternal deaths due to CVD are preventable^2^. In South Asia including Pakistan, the estimated prevalence of CVD in pregnancy is 1–2%^3^. However, this is likely an underestimation of the actual prevalence due to multiple factors such as the lack of complete antenatal care, underdiagnosis of CVD in pregnancy, and substandard reporting of maternal death.

Although good-quality local and robust research data are lacking in developing countries, the existing literature supports the fact that a third of maternal deaths occurring in the intrapartum and postpartum period are CVD-related^2^. Most of the present data are on the incidence of CVD in pregnancy and great paucity is observed in data on prevalence, especially from the developing world^4^. According to the Global Burden of Disease (GBD) 2017 findings, there has been a significant decline globally in direct maternal deaths, however maternal deaths from indirect causes especially hypertensive disorder during pregnancy remains unchanged^5^. The GBD report also shows a wide range of maternal mortality rates (MMR) across the globe (from 1- 496 per 100,000 live births).

Pakistan is a developing country in South Asia with an estimated population of 224.1 million and a fertility rate of 3.7 per women based on the GBD 2017 report. According to maternal newborn health registry data from 2010–2018, a maternal mortality ratio (MMR) of 327 per 100,0000 live births in Pakistan was observed; with CVD (hypertensive disorder leading the entity) identified as a risk factor for the increased mortality [RR 6.87 (5.05,9.34)] compared with those without CVD. According to a study conducted in different Pakistani sites to understand maternal mortality, Fikree FF et al.^6^ found that eclampsia and congestive heart failure were notable causes of death contributing to 15% and 10% of maternal deaths, respectively. Another single-center study conducted by Begum et al.^7^ reported eclampsia which is a complication of hypertension in pregnancy in up to 30% of their cohort. A retrospective study conducted in 2015 at a private institute in Pakistan showed a 21% maternal mortality rate in pregnant patients admitted to the ICU and heart diseases accounted for 32% of these deaths^8^. In recently reported prospective large registry from Pakistan that screened 15, 608 asymptomatic pregnant women with echocardiography, 569 (3.8%) had structural heart disease of which 338 (2.2%) had left ventricular systolic dysfunction, 188 (1.2%) had valvular heart disease and 68 (0.5%) had congenital heart disease^9^. In another study of 14, 275 consecutive pregnant women screened at two hospitals in Hyderabad, India, 353 (2.5%) were found to have a structural abnormality on echocardiography^10^. These data suggest a large undiagnosed burden of heart disease in pregnant women. The Madras Medical College Pregnancy and Cardiac Registry (M-PAC) from India prospectively enrolled 1029 pregnant women with known or newly diagnosed cardiovascular disease for 2.5 years in the registry to assess the fetal and maternal outcomes. Rheumatic heart disease (RHD) was the most common cardiac disease among the cohort (433/1029, 42%). Maternal cardiac adverse events occurred in 15% of the pregnancies (156/1029; 95% CI: 13.0–17.5), with heart failure being the most common adverse event. They reported an overall maternal mortality of 1.9% with the highest mortality observed in patients with prosthetic heart valves^11^. The National Maternal Death Surveillance Response System (MDSR) in Sri Lanka investigated maternal deaths during pregnancy between 2006 to 2018. Of the 2855 pregnancy related deaths reported to the MDSR, 1646 (57.7%) were confirmed maternal deaths. Of the confirmed maternal deaths, 17.5% (284/1646) were due to cardiac diseases complicating the pregnancy. Among deaths attributable to heart disease, RHD, CMP, and CHD were the leading causes with a reported percentage of 21% (60/284), 20% (59/284), and 12% (34/284) respectively. In their analysis, 19% of the heart disease related maternal deaths were in medically contraindicated pregnancies. In addition, of all the heart disease related deaths, 55.6% (151/284) were categorized as preventable deaths. The group therefore emphasized specialized care for the women with CVD and pregnancy^12^.

## Methodology

A literature review was conducted using PubMed and Scopus databases with keywords related to CVD in pregnancy in South Asian low- and middle-income countries (LMICs). The search terms included “pregnancy and CVD,” “pregnancy-related physiologic changes,” “clinical presentation,” “reported epidemiology,” and “health system challenges.” Evidence was extracted from published observational studies, registry data, systematic reviews, and meta-analyses that included populations from South Asian LMICs and were relevant to CVD in pregnancy. Findings from the shortlisted studies were reviewed by the author’s team, and relevant information was incorporated into this review.

In the following paragraphs, we discuss the various commonly occurring cardiovascular diseases in Pregnancy.

### Cardiovascular diseases in pregnancy

#### Hypertension in pregnancy and pre-eclampsia

Hypertension in pregnancy is diagnosed based on clinic blood pressure values of greater than or equal to 140 mmHg systolic and greater than or equal to 90 mmHg diastolic. The disease is categorized into four types. Pre-existent or chronic hypertension (preceding pregnancy or developing before 20 weeks of gestation), Gestational hypertension (develops at or after 20 weeks of gestation and resolves by the end of the postpartum period), Preeclampsia (gestational hypertension with significant proteinuria) and pre-existing chronic hypertension with superimposed preeclampsia. Hypertension is the most common cardiovascular disease in pregnancy and complicates up to 10% of pregnancies in developed countries. In developing countries, the prevalence of hypertension in pregnancy is similar and according to a prospective population-level analysis done in India, Pakistan, Mozambique, and Nigeria in 2019, a prevalence of 9.3% in Pakistan and 10.3% in India is reported^13^.The analysis includes 28, 420 pregnancies enrolled in Community Level Intervention for Pre-eclampsia Cluster Randomized Trial (CLIP) Trial. In this analysis, compared with pre-existing hypertension and pre/eclampsia, gestational hypertension was the most common diagnosis at first presentation with a frequency of 6.5% in Pakistan, 6.9% in India, 7.1% in Nigeria, and 8.4% in Mozambique^13^.

The point to ponder here is the long-term effects of hypertension on maternal and newborn health. It has been suggested that children born to pre-eclamptic mothers are at increased risk of developing conventional risk factors for cardiovascular diseases in their adulthood such as hypertension, and obesity. The future development of premature cardiovascular disease in adulthood is reported in children born to preeclamptic mothers^14^. Women who experience hypertension and preeclampsia during pregnancy, are at a 4-fold increase in risk of future incident of heart failure and a 2-fold increased risk of coronary heart disease later in life^15^. The physiological changes during pregnancy, compounded by endothelial dysfunction and vascular remodeling, contribute to this elevated long-term cardiovascular risk^16^. These findings underscore the importance of long-term cardiovascular monitoring and preventive strategies for women who have experienced hypertension and preeclampsia.

#### Rheumatic valvular heart disease

In developing countries Rheumatic heart disease (RHD) accounts for majority of the valvular heart disease (VHD) illnesses during pregnancy. Registry of Pregnancy and Cardiac Diseases (ROPAC) data of 2966 patients from 2008 to 2014 from both high- and low-income countries reported an RHD frequency of 13% and most patients were from developing countries. Recently published data of 1029 pregnant women from the M-PAC registry in India has reported a prevalence of 433/1029 (42%) RHD among the total study cohort^11^. Of those with RHD, mitral valve involvement was the most common with combined mitral stenosis and regurgitation noted in up to 30%, while isolated mitral stenosis and regurgitation was noted in up to 20.8% and 17.3% respectively. These patients showed relatively poor maternal and fetal outcomes compared to healthy mothers. In a study of pregnant women with RHD, preterm delivery occurred in 23.7% of cases, and 53.7% of fetuses had low birth weight^17^.

#### Congenital heart diseases

Maternal congenital heart diseases (CHD) are present in up to 1% of live births. The pregnancy outcome is dependent on the type and severity of the underlying CHD^1^. An increasing burden of CHD seen in pregnancy is possibly due to better care provided to CHD patients in childhood resulting in more women reaching to reproductive age^18^. In the prospective registry of pregnant women screened with echocardiography from Pakistan, 68 out of 15, 068 (0.5%) had congenital heart disease of which atrial septal defects, ventricular septal defects and tetralogy of Fallot were the top three most common congenital lesions^9^. In the cohort of echocardiographic screening of pregnant women from India, the most common congenital heart conditions included atrial septal defect, patent ductus arteriosus, and bicuspid aortic valve^10^.

#### Heart failure

Cardiomyopathy (CMP) and heart failure (HF) in pregnancy are common complications of existing or acquired heart diseases in pregnancy and result in higher maternal morbidity and mortality^19^. In Pakistan, 338/15,068 (2.2%) of asymptomatic women screened with routine echo during pregnancy had left ventricular systolic dysfunction of mild (41–55%), moderate (31–40%) and severe intensity (<30%)^7, 9^. But in the cohort from India, 66/14275 (0.4%) screened pregnant women had either dilated cardiomyopathy or left ventricular systolic dysfunction defined as an ejection fraction of less than 55%^10^. Data from the ROPAC registry focusing on heart failure as a primary outcome from 2007 to 2011 showed an HF incidence of 17% (173/1321 patients) among women with CVD in pregnancy. HF was associated with a maternal mortality of 4.8% compared to 0.5% in women without HF (*P* = < 0.001) and the highest incidence of HF was seen at the end of the second trimester. CHD, VHD, CMP, and pre-eclampsia were the most common etiologies of HF in ROPAC data.

#### Maternal mortality and CVD care in South Asia

According to WHO a moderate to higher maternal mortality ratio (MMR) is observed (545/100,000 live births in Sub-Saharan Africa to 129/100,000 live births in Southern Asia in 2020. Table 1 shows the country-wide MMR /100,000 live births in South Asia. Along with maternal hemorrhage and infection, hypertension, and pre-eclampsia are identified as major reasons behind preventable maternal deaths^3^. Table 2 shows the availability of national CVD societies, Multidisciplinary teams (MDT), and pregnancy specific national guidelines in South Asian LMICs.

**Table 1:** WHO 2023 country, region global health statics

Source of data	Country	^a^Maternal mortality ratio/100,000 live births
**WHO Annual World Health Statistics Report**	**Afghanistan**	**620**
	**Bangladesh**	**123**
	**Bhutan**	**60**
	**India**	**103**
	**Maldives**	**57**
	**Nepal**	**174**
	**Pakistan**	**154**
	**Sri Lanka**	**29**

^a^The table indicates the highest maternal mortality ratio in Afghanistan followed by Nepal and Pakistan.

**Table 2:** National CVD societies and disease-specific guidelines for pregnancy in South Asia

Country	National cardiology society	Multidisciplinary team involvement	Local guidelines			
			HTN	CHD	RHD	HF
**Afghanistan**	***✓***	**-**	**-**	**-**	**-**	**-**
**Bangladesh**	***✓***	**-**	***✓***	**-**	**-**	**-**
**Bhutan**	**-**	**-**	**-**	**-**	**-**	**-**
**India**	***✓***	**-**	***✓***	**-**	**-**	**-**
**Maldives**	**-**	**-**	***✓***	***✓***	***✓***	***✓***
**Nepal**	***✓***	**-**	***✓***	***✓***	***✓***	***✓***
**Pakistan**	***✓***	**✓**	**✓**	**-**	**-**	**-**
**Sri Lanka**	***✓***	**-**	***✓***	**-**	**-**	**-**

CVD=Cardiovascular, HTN=Hypertension, CHD=Congenital heart disease, RHD=Rheumatic heart disease, HF=Heart failure.

The **(*✓)*** in the table indicates the existence of a national cardiology society, multidisciplinary team involvement in the care of patients, and availability of local guidelines. The (-) indicates lack of these facilities.

### Potential reasons for high maternal mortality from CVD in South Asia with Pakistan as a use case

**Fig. 2:**
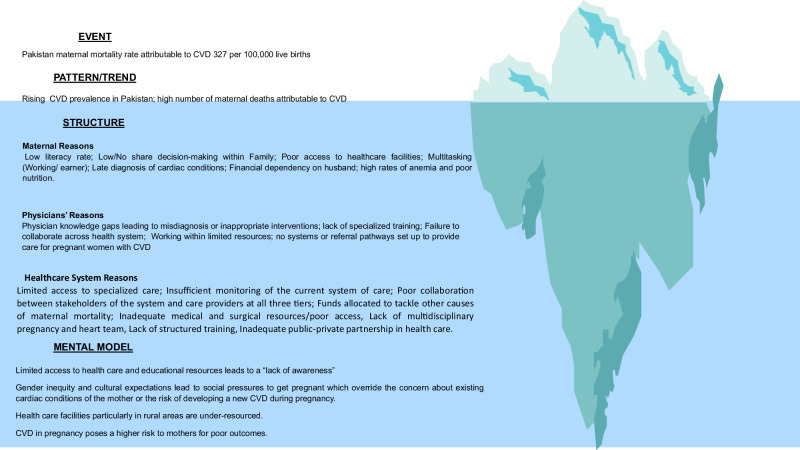
Iceberg model of system thinking for CVD in pregnancy. An illustration of the four levels of the systems thinking tool and the factors affecting the outcome of cardiovascular disease in pregnancy.

In the Fig. 2, we present potential reasons for high maternal mortality with CVD in Pakistan. We use the iceberg model to show the event, the patterns/trends, the underlying structures, and the mental model to dissect the potential multiple interrelated, multi-sectoral challenges that contribute to this problem. An iceberg model is a system thinking tool. The model is based on the idea of more factors involved in an underlying problem than what is initially apparent. The iceberg model structures our thinking around four levels. The first level is the event, which is the most visible part of the model. The event level is what people see and experience daily as a problem. There are typically many underlying factors that contribute to the problem. The next level is the pattern or trend, which represents the events happening over time or the factors anticipating the event. The third level is an underlying structure that is causing the pattern. The health system structure usually is comprised of clinicians, patients, and the health industry which includes hospitals and relevant stakeholders. Finally, is the mental model which includes the beliefs, assumptions, and principles that hold the system (see Supplementary Reference 1).

### Events

The event here that we are trying to understand is the high maternal mortality rate associated with cardiovascular disease during pregnancy. Cardiovascular diseases cause a significant risk to maternal health in low- and middle-income countries including Pakistan. A systematic review aimed to assess the frequency of maternal deaths due to cardiac diseases in low – and middle-income countries, incorporating 47 maternal mortality reports from 29 LMICs, excluding hospital-based and verbal autopsies-based reports. This review reported a total of 38,486 maternal deaths in LMICs. Out of these deaths, the cardiac disease-related maternal mortality ranged from 0-31/100,1000 live births^20^. LMICs with the highest maternal mortality rates also had the highest rates of cardiac-disease-related fatalities during pregnancy or childbirth^20^. Cardiac disease in the mother is also associated with poor outcomes for the fetus/newborn. An audit conducted on perinatal stillbirths at a public sector tertiary care hospital in Karachi, Pakistan in 2000 for one year on 7743 deliveries. Among these there were 753 perinatal deaths (589 stillbirths and 184 died in the first week post-delivery). The leading cause of stillbirths was attributed to hypertensive disease in the mother (180/569, 24%)^21^. Similarly, in a cross-sectional study of 52 consecutive pregnant patients with diagnosed cardiac diseases in Kharian Punjab, Pakistan from December 2017 – December 2018 cardiac events complicated 38.8% (20/52) of these pregnancies. Majority developed congestive cardiac failure (7/20, 35%) arrhythmia (5/20, 25%), pulmonary edema (2/20, 10%), thromboembolism (2/20, 10%) and required cardiac intervention during pregnancy (1/20, 5%)^22^.

### Pattern/Trend

Cardiovascular disease in pregnancy is not a well-known studied entity in Pakistan. While extensive research has been conducted on maternal mortality, there is a significant gap in studies focusing on cardiovascular disease and its impact on pregnancy and its outcomes. There is a substantial lack of data on the incidence of maternal deaths attributable to cardiac diseases in LMICs like Pakistan^20^. More robust research, such as prospective studies and implementation research on CVD in pregnancy, needs to be undertaken. Additionally, there is a lack of structured multidisciplinary pregnancy heart teams in most of the public and most private healthcare setups.

### Structure

Several factors contribute to the high maternal mortality rate in low- and middle-income countries like Pakistan.

We describe the structures contributing to poor outcomes in pregnant women with CVD through three different lenses: Maternal factors, clinician factors, and healthcare system-level factors.

#### Maternal Factors

Maternal factors contributing to high maternal mortality with CVD in pregnancy include low empowerment indices in women in Pakistan. As pointed out earlier, many pregnancies with adverse outcomes are, to begin with, contraindicated. Access to family planning and decision-making regarding pregnancy are key challenges in Pakistan. According to a report published in 2018 by the Pakistan Demographic and Health Survey (PDHS) only 34% of women in Pakistan use family planning (urban=43%, rural =29%). The report identified a higher gap in service availability and readiness at rural health centers (RHC) and basic health units (BHU). Only 58% of the centers offer an antenatal care service and only 40% monitor hypertensive disorders of pregnancy^23^. In addition to this low literacy and low health literacy rates contribute to low awareness of ideal care-seeking behavior in pregnancy. Antenatal care is essential for monitoring the health of pregnant women and identifying and treating any potential complications. The coverage and quality of antenatal care services in Pakistan vary significantly. While some women receive regular check-ups and necessary interventions, others may have limited access to adequate antenatal care, leading to missed opportunities for early detection and management of complications. Female mobility, purchasing power, and transport are some of the factors limiting access. It alone to seek care, 30% reported finance as a barrier, and 21% reported lack of permission to go to the hospital as a reason to delay health care access (https://www.countdown2030.org/wp-content/uploads/2021/02/Pakistan-CD-report-2020.pdf, *hereafter ‘Countdown 2020’*). Socio-cultural factors, including gender inequality, lack of awareness, and cultural practices, also influence women’s access to utilization of maternal health services in developing countries including Pakistan^3^.

Delay in seeking care is one of the potential causes of maternal mortality in Pakistan due to certain social and cultural factors. The contributing factors to delay in seeking antenatal care include a lack of knowledge regarding the importance of antenatal care, poor socioeconomic status, lack of decision-making power, and financial constraints^24^.Lack of education, particularly among women, and malnutrition-causing anemia are some of the other major factors contributing to high maternal mortality and pregnancy complications in LMICs. In specific cultural settings, pregnancy complications or danger signs are often seen as usual rather than indicating a need for medical intervention^25^.

#### Provider related factors

Healthcare professionals in low and middle-income countries, particularly in rural areas, are oblivious to the impact of cardiovascular disease in pregnancy, and lack of awareness leads to inappropriate care and preventable deaths^11^. Obstetric care guidelines emphasize the care of women with high-risk maternal conditions such as cardiovascular disease in centers where available resources and personnel are trained and capable of anticipating and providing their specific needs^26^. Recent guidelines outline the structure of pregnancy heart team and emphasize on use of modified World Health Organization risk stratification criteria for pregnant women^27,28^. In addition to the lack of awareness and training, number of healthcare professionals, including physicians, nurses, and Lady Health Visitors (LHVs), are often low, especially in rural areas. The doctor-to-patient ratio in Pakistan is 1:1,300, which is significantly less than the World Health Organization’s recommended ratio of 1:1000^29^. This shortage not only affects the availability of trained professionals but also the quality of care provided.

#### Healthcare system level factors

Pregnancy complicated by CVD requires timely access to emergency obstetric care for saving the lives of mothers and newborns. However, many healthcare facilities, particularly in remote areas, lack the necessary infrastructure, equipment, and skilled staff to provide emergency obstetric services, leading to delays in receiving care. In many LMICs, women do not have access to proper healthcare facilities like diagnostic tests and clinicians to diagnose cardiac disorders^20^. A population-based prospective cohort study conducted in rural districts of Pakistan between 2016 and 2017 reported on a total of 7572 pregnancies and their outcomes. It was found that maternal mortality was three times higher among women who were attended by unskilled birth attendants compared to those attended by skilled healthcare professionals^30^. In rural areas of Pakistan, scarcity of healthcare facilities, limited access to high risk obstetric care facilities, and poor infrastructure contribute to higher mortality rates compared to urban areas of Pakistan^5^. The current health system infrastructure for maternal care in Pakistan faces several challenges. While efforts have been made to improve maternal health services, significant gaps and limitations still need to be addressed. Healthcare facilities in Pakistan are a mix of public and private facilities, including hospitals, clinics, and maternity centers. However, there is a significant disparity in access to quality maternal care services between rural and urban areas. Urban areas generally have better-equipped facilities with more skilled healthcare providers^1^. In addition, the quality of training and skills among healthcare providers may vary, affecting the overall quality of maternal care.

To address these challenges, the Pakistani government, along with international partners, is working to improve maternal health services through policy reforms, capacity building, and infrastructure development. Efforts are being made to increase the availability and distribution of skilled healthcare providers, enhance access to quality antenatal and emergency obstetric care, and strengthen health information systems for better monitoring and evaluation. *(Countdown 2020).*

### Mental model

Lack of formal education among women results in poor awareness about proper antenatal care, pregnancy complications, ignoring high risk sign and symptoms during pregnancy, and the importance of skilled health care providers.

Cultural expectations and gender inequity lead to social pressures to get pregnant, which override concerns about the existing cardiac condition of the mother or the risk of developing a new CVD during pregnancy.

The scarcity of healthcare facilities, including diagnostic facilities and skilled healthcare professionals, has led to delays in diagnosing cardiovascular diseases during pregnancy.

The proposed solutions aim to address the critical challenges identified by the iceberg model in maternal healthcare, particularly in reducing maternal mortality and improving care for pregnant women with CVD. Ensuring timely access to emergency obstetric care and robust health information systems, especially in remote areas, can significantly reduce maternal mortality rates. Expanding access to antenatal care through awareness campaigns, financial support, and cultural shifts can empower women to seek timely medical attention, significantly improving maternal outcomes. For pregnant women with cardiovascular diseases (CVD), specialized care pathways, pre-conception risk assessments, and the availability of trained healthcare professionals are crucial. Similarly, investing in research on CVD in pregnancy and fostering international collaborations will provide valuable insights to inform policy and practice, ultimately improving maternal and neonatal health outcomes in developing regions. Additionally, strengthening civil registration and vital statistics systems can provide real-time, accurate maternal mortality data, enabling timely, area-specific interventions. Improved data infrastructure supports better monitoring and targeted policy actions, which are essential for reducing deaths in pregnant women with CVD. In Table 3 we summarize the challenges and offer possible solutions.

**Table 3:** Identified problems in the care system and possible solutions

Problem	Possible solution
High maternal mortality rates	Enhance antenatal care coverage, addressing barriers such as female mobility, financial constraints, and cultural factors. Implement and enforce policies to ensure timely access to emergency obstetric care, especially in remote areas. Strengthen health information systems for better monitoring and evaluation of maternal health services.
Lack of specialized care for cardiovascular diseases (CVD) in pregnancy	Establish specialized care pathways for pregnant women with CVD, including pre-conception risk assessments. Increase the availability and distribution of skilled healthcare providers trained in managing cardiovascular complications during pregnancy. Promote collaboration between healthcare team members involved in caring for pregnant women with CVD.
Disparities in healthcare infrastructure	Address the urban-rural gap by investing in better-equipped facilities in rural areas and ensuring a higher concentration of skilled healthcare providers.
Limited access to antenatal care	Improve female mobility through awareness campaigns and community engagement. Address financial barriers by implementing subsidized or free antenatal care services. Challenge cultural norms restricting women’s access to healthcare and promote gender equality.
Limited research and data on CVD in pregnancy in developing countries	Allocate resources for research focused on CVD in pregnancy in developing countries and encourage international collaborations and partnerships to share knowledge and expertise in addressing CVD in pregnancy.

The table summarizes the identified problems in the care system at various levels, with a possible solution listed against each.

## Conclusion

We conclude that existing data highlight higher maternal mortality and morbidity in women with cardiovascular disease (CVD) during pregnancy in developing countries of South Asia. Hypertension and its related disorders in pregnancy are leading causes. Preconception counseling, along with improved access to antenatal care, is crucial in assessing and managing the maternal risks in women with pre-existing or high-risk cardiac conditions before and during pregnancy. Early identification of high-risk women and providing specialized care through a multidisciplinary pregnancy heart team is essential for improving outcomes. A coordinated effort between healthcare professionals is critical to ensure the best possible care for these women. Further research is needed in low- and middle-income countries to develop and implement context-specific interventions to address the specific needs at various levels of care described in the iceberg model.

## Supplementary information

Supplementary File

## Data Availability

No datasets were generated or analysed during the current study.
