# Supplementary material for: Pregnancy and cardiovascular disease in developing countries of South Asia—A narrative review
Source: NPJ Womens Health. 2025 Oct 7;3(1):56. doi: 10.1038/s44294-025-00101-y (PMC12504106; doi:10.1038/s44294-025-00101-y)
Supplement: Supplementary file 1 — Supplementary File [file 44294_2025_101_MOESM1_ESM.docx]

**Supplementary Reference 1**
Ecochallenge.org. *Iceberg Model*. Available at: https://ecochallenge.org/iceberg-model
